# Prevalence of autism in mainland China, Hong Kong and Taiwan: a systematic review and meta-analysis

**DOI:** 10.1186/2040-2392-4-7

**Published:** 2013-04-09

**Authors:** Xiang Sun, Carrie Allison, Fiona E Matthews, Stephen J Sharp, Bonnie Auyeung, Simon Baron-Cohen, Carol Brayne

**Affiliations:** 1 Department of Public Health and Primary Care, Institute of Public Health, University of Cambridge, Forvie Site, Robinson Way, Cambridge, CB2 0SR, UK; 2 Department of Psychiatry, Autism Research Centre, University of Cambridge, Douglas House, 18b Trumpington Road, Cambridge, CB2 2AH, UK; 3 MRC Biostatistics Unit, Institute of Public Health, Forvie Site, Robinson Way, Cambridge, CB2 0SR, UK; 4 MRC Epidemiology Unit, Institute of Metabolic Science, Addenbrooke’s Hospital, Hills Road, Cambridge, CB2 0QQ, UK

**Keywords:** Autism spectrum conditions, Prevalence, Screening, Diagnosis, Chinese population

## Abstract

**Background:**

The prevalence of autism spectrum conditions (ASC) is 1% in developed countries, but little data are available from mainland China, Hong Kong and Taiwan. This study synthesizes evidence relating to the prevalence of ASC in these areas and assesses the effects of research methodology on prevalence estimates.

**Methods:**

Systematic literature searches were conducted in PubMed, Web of Knowledge, China Web of Knowledge and Weipu databases, as well as relevant papers published from 1987 to 2011, reporting prevalence estimates of ASC or childhood autism in mainland China, Hong Kong and Taiwan. Summary estimates of prevalence were calculated with a random effects model. The effects of research methodology on the prevalence estimates were assessed using a meta-regression model.

**Results:**

There were 25 studies eligible for review, 18 of which were suitable for inclusion in a meta-analysis. Pooled prevalence of childhood autism was 11.8 per 10,000 individuals (95% confidence interval (CI): 8.2, 15.3) in mainland China. Pooled prevalence of ASC was 26.6 per 10,000 (95% CI: 18.5, 34.6) in three areas. Substantial heterogeneity was identified between studies (*I*^2^>75%). The prevalence estimate of childhood autism was most strongly associated with the choice of screening instrument. After adjustment for age group, the odds ratio for prevalence estimates when using the Autism Behavior Checklist (ABC) as the screening instrument compared with those using the Clancy Autism Behavior Scale (CABS) was 0.29 (95% CI: 0.12, 0.69), and 1.79 (95% CI: 0.70, 4.55; *P*= 0.20) when using the Checklist for Autism in Toddlers (CHAT) compared to the CABS.

**Conclusions:**

The available studies investigating the prevalence of ASC in China, Hong Kong and Taiwan have focused mainly on childhood autism rather than the whole spectrum. The prevalence estimates are lower than estimates from developed countries. Studies using more recently developed screening instruments reported higher prevalence than older ones. However, available studies have methodological weaknesses and therefore these results lack comparability with those from developed countries. Our findings indicate a potential under-diagnosis and under-detection of ASC in mainland China, Hong Kong and Taiwan, and a need to adopt more advanced methods for research of ASC in these areas.

## Review

### Introduction

Autism spectrum conditions (ASC) are characterized by impairments in social interaction and communication, and the presence of repetitive and stereotyped behaviors, interests and activities, and these impairments are present during the life-course [1,2]. ASC are considered to have a substantial functional and financial impact on affected individuals and their families [3,4]. The first prevalence estimate of autism, as described by Leo Kanner, was 4.5 per 10,000 people among children aged 8 to 10 in the southeast of England [5]. There has been a rise in prevalence estimates reported in population-based studies in developed countries. The most recent prevalence estimate of ASC in the UK was 157 per 10,000 in 2009 [6] and 113 per 10,000 in the US in 2012 [7]. In the East, studies in Japanese populations showed that the prevalence of ASC increased from 21.1 per 10,000 in 1996 [8] to 181 per 10,000 in 2008 [9]. A recent study in South Korea reported a prevalence estimate of 264 per 10,000 in 2011 [10].

**Table 1:** Summary of prevalence studies of autism spectrum conditions in mainland China, Hong Kong and Taiwan (25 studies)

**Year**	**First author**	**Region**	**Sample size**	**Area**	**Age (years)**	**Sample screened**	**Screen methods**	**Screen tools**	**Cut-off**	**Response rate (%)**	**P/R**	**Diagnostic tools**	**Diagnostic criteria**	**Childhood autism**	**ASC prevalence/SE (per 10,000)**
														**prevalence/SE (per 10,000)**	
1987	Tao [37]	Mainland	457,200	Urban	3 to 8	C	R	-	-	-	R	-	Rutter	0.32 (0.08)	-
2000	Luo [38]	Mainland	10,802	Mixed	2 to 14	SG	QI	ABC	31	100	P	-	CCMD-2-R, DSM-III-R	2.8 (1.60)	-
2002	Wang [39]	Mainland	3.978	Urban	2 to 6	K	QI	CABS	7	98.3	P	CARS	CCMD-2-R	17.9 (6.70)	-
2002	Ren [40]	Mainland	3,559	Urban	3 to 5	SG	QI	CABS	14	99.1	P	-	-	250 (2.31)	-
2003	Wang [41]	Mainland	7,488	Mixed	2 to 6	SG	QI	CABS	7	98.08	P	CARS	CCMD-2-R	12.3 (4.05)	-
2003	Chang [42]	Taiwan	660	Mixed	15 to 93	C	C	ASDASQ	5	100	P	-	DSM-IV	-	60.0 (30.06)
2004	Guo [43]	Mainland	5,000	Urban	0 to 6	WP	QI	CABS	7	99.1	P	CARS	CCMD-2-R	10 (4.47)	-
2004	Guo [44]	Mainland	3,776	Rural	2 to 6	SG	QI	CABS	7	100	P	CARS	DSM-IV	8 (4.59)	-
2005	Zhang [45]	Mainland	7,416	Urban	2 to 6	SG	QI	CABS	7	99	P	CARS	DSM-IV	11.0 (3.85)	-
2005	Zhang [46]	Mainland	1,305	Urban	3 to 7	K	QI	CABS	14	100	P	-	-	19.9 (2.47)	-
2005	Liu [47]	Mainland	21,866	Mixed	2 to 6	SG	QI	CABS	7	100	P	CARS	DSM-IV	13.4 (2.47)	15.3 (2.64)
2007	Yang [48]	Mainland	10,412	Urban	3 to 12	PS	QI	ABC	31	100	P	-	DSM-IV	5.6 (2.32)	-
2007	Wong [49]	Hong Kong	4,247,206	Mixed	0 to 14	HS	R	-	-	-	R	CARS, ADI-R	DSM-IV	-	16.1 (0.19)
2008	Zhang [36]	Mainland	8,681	Urban	2 to 3	SG	QI	CHAT	-	100	P	CARS	DSM-IV	16.1 (4.3)	-
2008	Zhang [36]	Mainland	12,430	Urban	4 to 6	SG	QI	CABS	14	100	P	CARS	DSM-IV	8.85 (2.7)	-
2009	Zhang [50]	Mainland	5,000	Urban	0 to 6	SG	QI	CABS	7	99.98	P	CARS	CCMD-2-R	10.0 (4.47)	-
2009	Wang [51]	Mainland	4,156	Urban	2 to 6	K	QI	CABS	14	100	P	-	-	19.5 (6.84)	-
2010	Li [52]	Mainland	8,006	Mixed	1.5 to 3	SG	QI	CHAT	-	92.99	P	CARS	DSM-IV	26.2 (5.71)	-
2010	Wu [53]	Mainland	8,532	Urban	0 to 3	SG	QI	CHAT	-	100	P	CARS	DSM-IV	8.2 (3.10)	-
2010	Yu [54]	Mainland	7,059	Mixed	2 to 6	SG	Q	CABS	7	89.7	P	-	DSM-IV	21.2 (5.47)	22.7 (5.66)
2010	Chen [55]	Mainland	7,034	Mixed	2 to 6	SG	Q	CABS	7	98.78	P	CARS	DSM-IV	14.2 (4.49)	24.2 (5.86)
2011	Wang [56]	Mainland	7,500	Urban	2 to 6	K	QI	CABS	14	87.8	P	-	DSM-IV	29.5 (6.26)	75.4 (9.99)
2011	Liang [57]	Mainland	2,485	Urban	3 to 6	K	QI	CABS	14	100	P	-	DSM-IV, ICD-10	14.1 (7.53)	-
2011	Li [58]	Mainland	616,940	Mixed	0 to 17	SG	QI	ABC	-	-	P	-	ICD-10	2.38 (0.20)	-
2011	Chien [59]	Taiwan	372,642	Mixed	0 to 17	HS	R	-	-	-	R	-	ICD-9	-	28.7 (0.88)

Sample screened: C, clinical patients; HS, population in health system; K, kindergartens; PS, primary schools; SG, stratified general population; WP, whole population. Screen methods: C, clinical referral; Q, questionnaire distribution; QI, questionnaire-based interview; R, records. Screen tools: ABC, Autism Behavior Checklist; ASDASQ, Autism Spectrum Disorder in Adults Screening Questionnaire; CABS, Clancy Autism Behavior Scale; CARS, Childhood Autism Rating Scale; CHAT, Checklist for Autism in Toddlers. P, Perspective; R, Retrospective. Diagnostic criteria: ADI-R, Autism Diagnostic Interview-Revised; CCMD-2-R,Chinese Classification of Mental Disorders, 2nd edition, revised; DSM-III-R, Diagnostic and Statistical Manual of Mental Disorders, 3rd edition, revised; DSM-IV, Diagnostic and Statistical Manual of Mental Disorders, 4th edition; ICD-9, International Classification of Diseases, 9th revision; ICD-10, International Classification of Diseases, 10th revision.ASC, autism spectrum conditions; SE,: Standard error; -, data not available**.**

Several reasons have been proposed to the current high prevalence, aside from a real increase: 1. the definition of autism has become broader [11]; 2. changes in diagnostic criteria and possible diagnostic substitution [12]; 3. changes in screening and diagnostic instruments for case ascertainment [13]; 4. changes in research methodology [14]; and 5. greater awareness and recognition of ASC [15].

In the UK, the National Autism Plan for Children (NAPC) was issued in 2003 [16], which listed strategies for identification, assessment, diagnosis and access to early intervention for preschool and primary school children with ASC. In clinical settings, there are several stages for diagnosing a child with suspected ASC. When the child is being seen by a general practitioner for a developmental assessment, the following examinations should be included: 1. developmental history; 2. physical examination; and 3. necessary and appropriate medical investigations according to the clinical presentation of the child. If during these examinations a diagnosis of ASC is suspected, the child should be recommended to the next stage, which is a multidisciplinary multiagency assessment (MAA) [17]. The NAPC also recommended that at least one team member should be trained in using a standardized assessment tool, the Autism Diagnostic Observation Schedule [18] (ADOS) or the Autism Diagnostic Interview-Revised [19] (ADI-R).

In East Asia, autism was not recognized by researchers until the early 1980s in mainland China [20,21]. Mainland China has a large population of over 1.37 billion, however, little is known about the extent of ASC in this country. One published review [22] estimated that the prevalence of childhood autism was 10.3 per 10,000 in mainland China, based on eight epidemiological studies. A recent study reviewed the instruments used for case identification of ASC in mainland China [23]. It indicated that the Clancy Autism Behavior Scale (CABS) [24] and the Autism Behavior Checklist (ABC) [25] as screening instruments, and the Childhood Autism Rating Scale (CARS) [26] as a diagnostic instrument, were the most frequently used in mainland China. The CABS was developed in 1969 [24] and introduced into China in the late 1980s. There have been very few updates since it was first translated and validated. It has been widely used in epidemiological research on childhood autism in mainland China, however the data on its validity and reliability in the West is lacking. The CABS was first published in 1969 [24]. However, there is no literature focusing on the utility of the CABS in the Western population. The Chinese version of the CABS was designed to be completed by parents. It contains 14 items with each item rated on three frequency levels including ‘never (score 0)’, ‘occasionally (score 1)’ and ‘often (score 2)’. If the child scores equal to or higher than 14, and has less than 3 items score as ‘Never’ and more than 6 items as ‘Often’, then the child should be considered as a potential case of childhood autism.

The CARS and ABC were also adopted early and are still in use for ASC research in the West [27,28]. Similarly, little is known about ASC in Hong Kong and Taiwan. The prevalence estimates of ASC in China are much lower than those from developed countries. The identification of ASC depends on autistic features shown during social interaction and communication. The identified autistic features could be influenced by culture as different cultures have different behavioral norms and expectations [29]. The heterogeneity of behaviors among individuals with ASC may relate to different phenotypes of ASC [30]. Thus, studies focusing on behaviors related to ASC should take cultural influence into consideration, especially for cross-sectional studies, since there may be an association between culturally influenced behaviors and the genetic origin of different phenotypes of ASC [31]. Cultural factors could affect the prevalence estimates of the ASC which in turn could affect the directions of molecular genetic studies. In addition, previous studies have reported that the screening and diagnostic criteria used in mainland China may be different from Western studies [22,23]. For example, one of the diagnostic criteria used in mainland China is the Chinese Classification of Mental Disorders (CCMD). The CCMD categorizes autism as a childhood psychiatric condition, the diagnostic domains of which were similar to those in the Diagnostic and Statistical Manual of Mental Disorders (DSM) and the International Classification of Diseases (ICD). The CCMD-2 has been in use since 1993 [32], while the CCMD-3 was issued in 2001 (Additional file 1)[33]. This means that the CCMD has not kept track with changes in DSM.

The aims of this study are: 1. to identify all available studies on the prevalence of ASC in mainland China, Hong Kong and Taiwan; 2. to assess the quality of this research; and 3. to evaluate the effects of the chosen research methodology on the prevalence estimates.

## Method

### Search strategy and selection criteria

A systematic review using PubMed, Web of Knowledge, China Web of Knowledge and Weipu databases was undertaken to identify any study in each database published between 1987 to December 2011, in either English or Chinese, reporting prevalence estimates of ASC or childhood autism in mainland China, Hong Kong and Taiwan. A search strategy was developed, with a comprehensive list of terms (Additional file 2), including those relating to the condition (for example, ‘autism’, ‘autistic disorders’, ‘autism spectrum’, ‘pervasive developmental disorders’, ‘Asperger’), study type (for example, ‘prevalence’, ‘epidemiology’, ‘screening’) and location (for example, ‘China’, ‘Hong Kong’, ‘Taiwan’). In addition, the bibliographies of previous reviews were examined to identify published prevalence studies [1,22,34]. All searches were conducted twice. The literature search, data extraction and quality assessment was undertaken by the first author. Identified papers were examined against inclusion criteria (Additional file 3). All the abstracts were reviewed and duplicates excluded. When it was not clear from the abstract whether the paper should be included, the paper itself was examined where possible. When the papers were not available, the corresponding authors were contacted to obtain the papers. If a paper provided prevalence estimates for different age groups using different methods, it was reported as separate studies due to the adoption of different study methods.

### Data abstraction

Following the removal of duplicates, the following variables were extracted from each paper: sample characteristics (year of publication, sample age, sex, region, location), sampling strategy, screening methods, diagnostic criteria, response rate (of screening/diagnostic assessment) and prevalence estimation measures.

### Statistical analysis

Crude prevalence estimates, confidence intervals (CIs) and study details were extracted from each paper where available. The identified studies were divided into two groups based on diagnosis: 1. childhood autism, included all studies that had provided a prevalence estimate for childhood autism, or autistic disorder; and 2) ASC, included all studies that estimated the prevalence for the whole autism spectrum. Forest plots were drawn to visualize the extent of heterogeneity among studies.

A random effects meta-analysis was used to estimate the overall prevalence and investigate the heterogeneity between studies. CIs were calculated from the crude prevalence estimates if not available. The extent of heterogeneity was estimated by calculating *I*^2^ (values of 25%, 50% and 75%, representing low, medium and high heterogeneity, respectively) [35]. The proportion of between-study variance explained by the covariates was estimated using adjusted *R*^2^values. Meta-regression was used to estimate the effect of the following covariates on the log odds of the outcome: age group, year of publication, publication period, area, sample source, sample size, screening method, screening instrument, screening informants, diagnostic tool, diagnostic criteria and diagnostic informant. Each covariate was included separately and then a multivariable meta-regression model was constructed including all covariates with a statistically significant (*P* <0.05) association in the univariate analyses.

## Results

In the first search attempt, the literature search identified 196 papers after the first two steps and 12 papers were identified after Step 3 in PubMed. In the Web of Knowledge, the first two steps identified 83 papers and 13 were identified after Step 3. In total, 25 papers including duplicates were further examined against inclusion criteria. Seven papers met the inclusion criteria and were selected for this review. The second search attempt was conducted within the 196 papers in PubMed and 83 papers in Web of Knowledge, which assured the seven papers selected and no other papers had been missed.

Within the Weipu database, search Step 1 identified 2,028 papers and Step 2 identified 51 studies for further examination against inclusion criteria. Within the China Web of Knowledge, search Step 1 identified 3,919 papers and Step 2 identified 80 papers. Combining the papers from both Chinese databases, after removal of the duplicated papers, there were 15 papers in total identified according to the inclusion criteria for this review. The second search within the two Chinese databases was conducted to reassure the select results. Another four papers were further identified which were not in the first literature search result. In total, 19 papers were identified from Chinese databases. In parallel, eight studies were identified from previous reviews.

**Figure 1:**
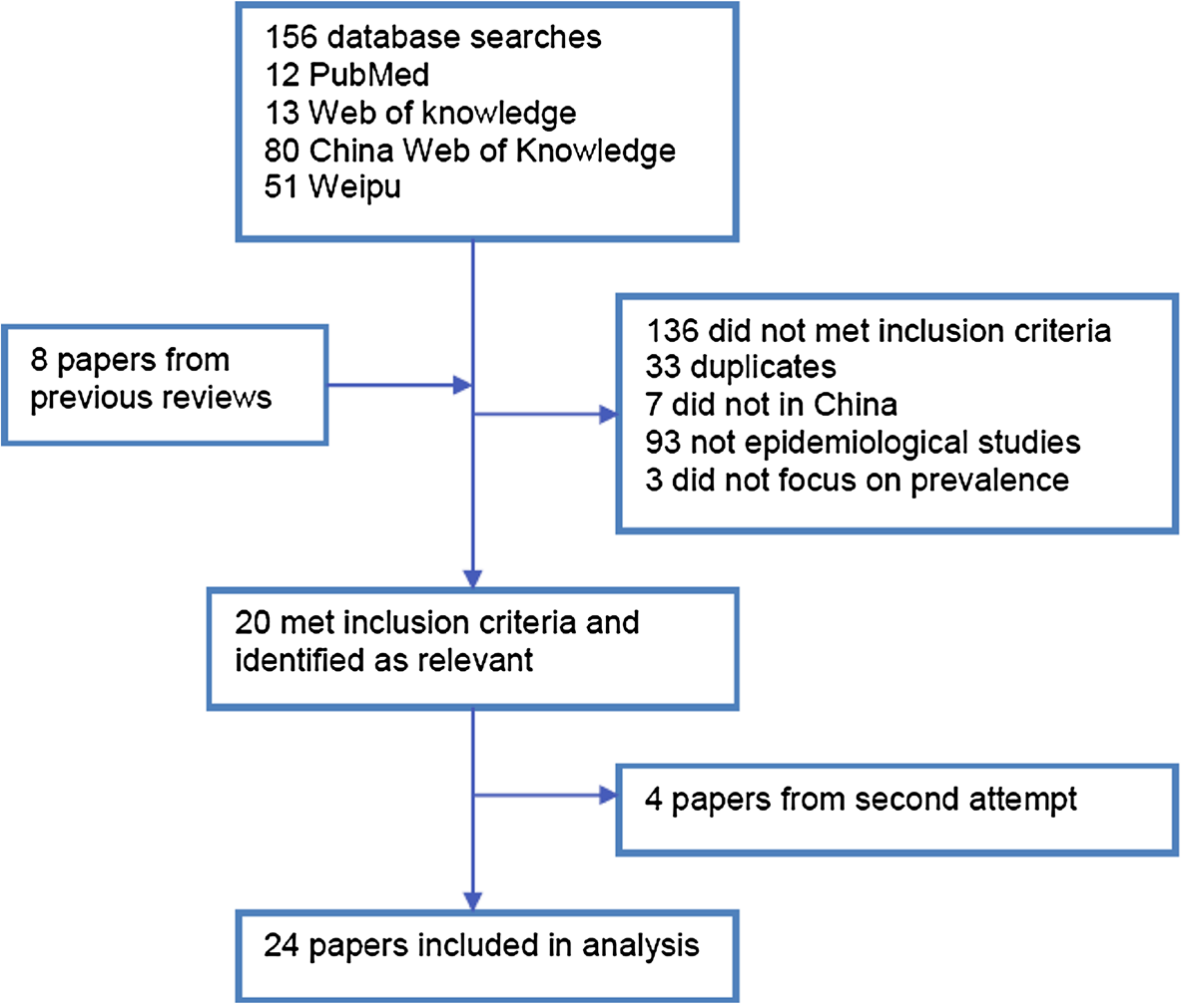
**Flowchart for selection of studies.** Description of search process and results of the four databases.

After the removal of duplicates between Chinese and English databases, a total of 25 studies were identified for analyses (Figure 1). One paper reported two prevalence studies within two different age groups using different methodologies. This paper was considered as two studies in the following analyses [36]. There were 21 studies conducted in mainland China, two studies in Taiwan and one study in Hong Kong (Table 1). In all of the studies, information about other minority populations other than the Chinese population was generally unavailable.

**Table 2:** Results of meta-regression childhood autism (18 studies)

**Univariate analyses**						
**Covariate**	**Categories of covariate**	**Number of studies**	**Odds ratio**	**95%CI**	***P *value**	**Variance explained (%)**
No covariates		18				
Year (continuous)		18	1.04	(0.93, 1.17)	0.43	−5.09
Year (categorical)	2000 to 2004	5	1.00	-	-	−10.92
	2005 to 2009	6	1.13	(0.42, 3.08)	0.80	
	2010 to 2011	7	1.44	(0.55, 3.77)	0.43	
Age group	<4	5	1.00	-	-	56.00
	4	8	1.06	(0.56, 2.02)	0.84	
	>4	5	0.33	(0.16, 0.68)	0.005	
Area	Urban	9	1.00	-	-	−8.40
	Mixed or rural	8	0.81	(0.38, 1.75)	0.57	
	Rural	1	0.64	(0.09, 4.37)	0.62	
Sample group	≤5000	5	1.00	-	-	7.43
	5000 to 7500	5	1.43	(0.54, 3.80)	0.44	
	>7500	8	0.74	(0.30, 1.80)	0.48	
Sample source	Population-based	13	1.00	1.00		−0.19
	Schools or kindergartens	4	1.44	(0.60, 3.48)	0.39	
Screening method	Interview	15	1.00	-	-	−0.89
	Questionnaire	2	1.64	(0.54, 4.98)	0.36	
Screening tool	CABS	12	1.00	-		76.99
	ABC	3	0.21	(0.11, 0.38)	<0.001	
	CHAT	3	1.25	(0.71, 2.20)	0.42	
Time	Once	13	1.00	1.00		−2.10
	Twice	4	1.40	(0.60, 3.27)	0.41	
Screening informant	Clinician	8	1.00	-	-	−13.86
	Parent	3	0.93	(0.31, 2.75)	0.88	
	Research	6	0.90	(0.38, 2.10)	0.79	
Diagnostic criteria	CCMD-2-R	5	1.00	-	-	−5.34
	DSM-III-R/DSM-IV/ICD-10	12	1.23	(0.52, 2.87)	0.62	
Diagnostic tool	None	6	1.00	-		2.54
	CABS	11	1.51	(0.71, 3.21)	0.27	
Diagnostic informant	Clinician	13	1.00	-	-	8.19
	Researcher	4	0.47	(0.19, 1.17)	0.10	
**Multivariable analyses**						
Age group	<4	5	1.00	-	-	80.7
	4	8	1.61	(0.68, 3.81)	0.26	
	>4	5	1.03	(0.34, 3.04)	0.96	
Screening tool	CABS	12	1.00	1.00	-	
	ABC	3	0.29	(0.12, 0.69)	0.009	
	CHAT	3	1.79	(0.70, 4.55)	0.20	

ABC, Autism Behavior Checklist; CABS, Clancy Autism Behavior Scale; CCMD-2-R, Chinese Classification of Mental Disorders, 2nd edition, revised; CHAT, Checklist for Autism in Toddlers; CI, confidence interval; SM-III-R, Diagnostic and Statistical Manual of Mental Disorders, 3rd edition, revised; DSM-IV, Diagnostic and Statistical Manual of Mental Disorders, 4th edition; ICD-10, International Classification of Diseases, 10th revision;-, data not available.

The population size of the reviewed studies ranged from 660 to 4,247,206 with a median sample size of 7,238 people. Two studies aimed to represent the whole area of Hong Kong [49] and Taiwan [59], while one study was generated from the national survey for disability in mainland China [58]. The age of participants included in the studies mainly ranged from 0 to 17 years, of which 23 studies focused on children aged 0 to 6 years and 12 studies on children aged 6 to 14 years (Additional file 4).

There were four sample sources: 1. clinical case counting of outpatients referred to psychological hospitals, two studies [37,42]; 2.case counting from hospital records in the national health system, two studies [49,59]; 3. random selection from local kindergartens, five studies [39,46,51,56] and one sample was from primary schools [48]; and 4.random selection from the general population, 14 studies selected using a two-stage approach. The first stage was stratification of the general population followed by randomized sampling from the stratified sample. There were five methods of sampling including case counting, randomized sampling, whole sample, clustered probability sampling and cluster-randomized sampling (Additional file 5).

Other than three studies which did not conduct screening but instead identified cases from existing health records [37,49,59], screening was conducted using the following approaches: 1. prospective screening in clinics (n = 1) [42]; 2.face-to-face interviews with a questionnaire (n = 19); 3.postal questionnaires (n = 1) [55]; and 4.postal questionnaires followed by face-to-face interviews (n = 1) [54].

Five studies conducted a second screening phase, while 17 studies only conducted one screening phase. Within the first screening phase, four instruments were used including the CABS (n = 15) [24], the ABC (n = 3)[60], the Checklist for Autism in Toddlers (CHAT) (n = 3) [13] and the Autism Spectrum Disorder in Adults Screening Questionnaire (ASDASQ) (n = 1) [61]. Three studies used the ABC and two used the CARS as screening tools after the first screening phase [26].

In the diagnostic phase, studies generally did not provide details about the diagnostic procedure. Three studies considered the screening results to be the final diagnosis without any additional diagnostic assessment [37,49,59]. Twelve studies reported that the diagnosis was made according to clinical judgment using international diagnostic criteria without using any diagnostic instrument [37,38,40,42,46,48,51,54],[56-59]. Twelve studies adopted the CARS as the diagnostic instrument [36,39,41,43-45,50,52,53,55],[62], and one study adopted both the CARS and the ADI-R [49,63]. Thirteen studies confirmed the diagnosis by clinicians conducting an interview with the parents or caregivers, of which 11 studies reported the interrater agreement between the clinicians using the kappa measure of agreement [36,38,39,43-45,50,52,53,55],[62], while the others used percentage agreement (Additional file 6).

Six different types of diagnostic criteria were used for case ascertainment which determined a prevalence estimate for autism: 1. Rutter’s criteria (n = 1); 2. Chinese Classification of Mental Disorders, 2nd edition, revised (CCMD-2-R) (n = 5) [32]; 3. Diagnostic and Statistical Manual of Mental Disorders, 3rd edition, revised (DSM-III-R) (n = 1) [64]; 4. DSM-IV (n = 13) [65]; 5. International Classification of Diseases, 10th revision (ICD-10) (n = 2) [2]; and 6.International Classification of Diseases, 9th revision (ICD-9) (n = 1).

Twenty-one studies reported response rates at the screening phase. In the reviewed prospective studies, participation rates in the diagnostic phase were not directly reported, since these studies conducted the screening and diagnosis during a single appointment. In these cases, when the child scored above the cut-off on the screening instrument, the diagnostic assessment was conducted immediately. Therefore, these studies only reported the final participation rate following the diagnostic phase. Where no information was provided about the children who were not assessed in the diagnostic phase, the participation rate was assumed to be 100%, which assumes that all children who screened positive completed a further diagnostic assessment during the analysis.

**Figure 2:**
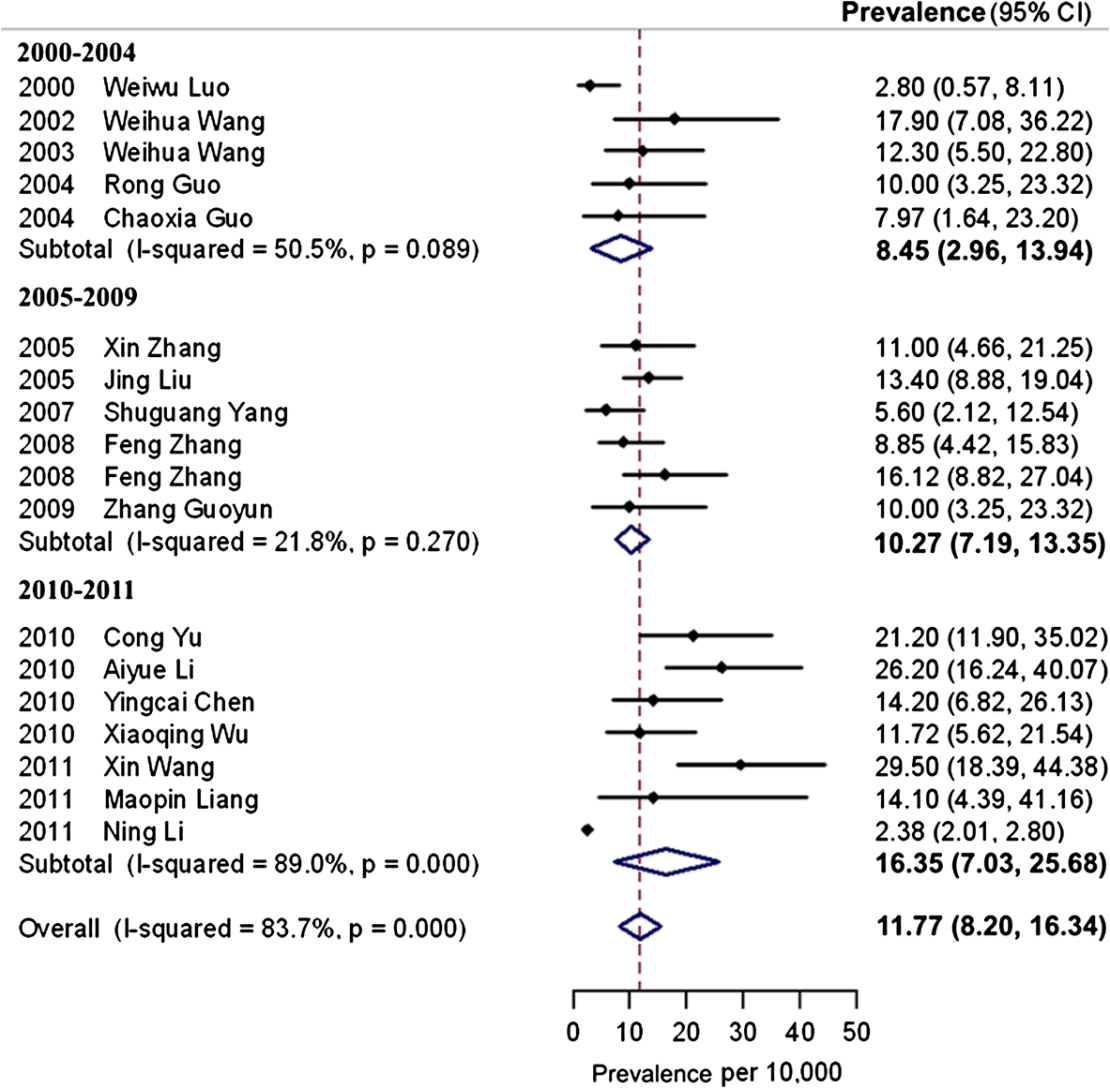
**Prevalence of childhood autism (n = 18).** Dots indicate prevalence estimates in reviewed studies. Horizontal lines indicate 95% CIs. Vertical line indicates pooled prevalence estimate in the meta-analysis. The heterogeneity of 18 studies was high (*I*^2^ = 83.7%). Prevalence estimates were presented for each reviewed study on childhood autism with 95% CIs. Reviewed studies were divided into three groups according to publication period: 2000 to 2004, 2005 to 2009 and 2010 to 2011. The pooled prevalence estimates for all reviewed studies and studies in each period were generated by a random effect meta-analysis model. There is an increase in the pooled prevalence estimates of childhood autism over time. CIs, confidence intervals.

Twenty-two studies provided prevalence estimates for childhood autism. Seven studies provided prevalence estimates for ASC (Additional file 7), of which four studies also investigated the prevalence of other subtypes, including atypical autism and pervasive developmental disorder not otherwise specified (PDD-NOS) [54-56,62]. Eighteen studies conducted both screening and diagnostic assessments for identifying cases of childhood autism in mainland China (Figure 2). The pooled prevalence estimate for childhood autism among these 18 studies was 11.8 per 10,000 (95% CI: 8.2, 15.3). The pooled prevalence estimate for ASC was 26.6 per 10,000 (95% CI: 18.5, 34.6).

The heterogeneity of the prevalence estimates among the 22 studies on childhood autism was very high (*I*^2^ = 93.4%). In further analyses, four studies on childhood autism were excluded since one was case counting of hospital records and three only conducted screening without a diagnostic phase [40,46,51]. The heterogeneity of 18 studies was reduced but still high (*I*^2^ = 83.7%). The heterogeneity of the seven studies describing the prevalence of ASC was also high (*I*^2^ = 97.4%).

**Figure 3:**
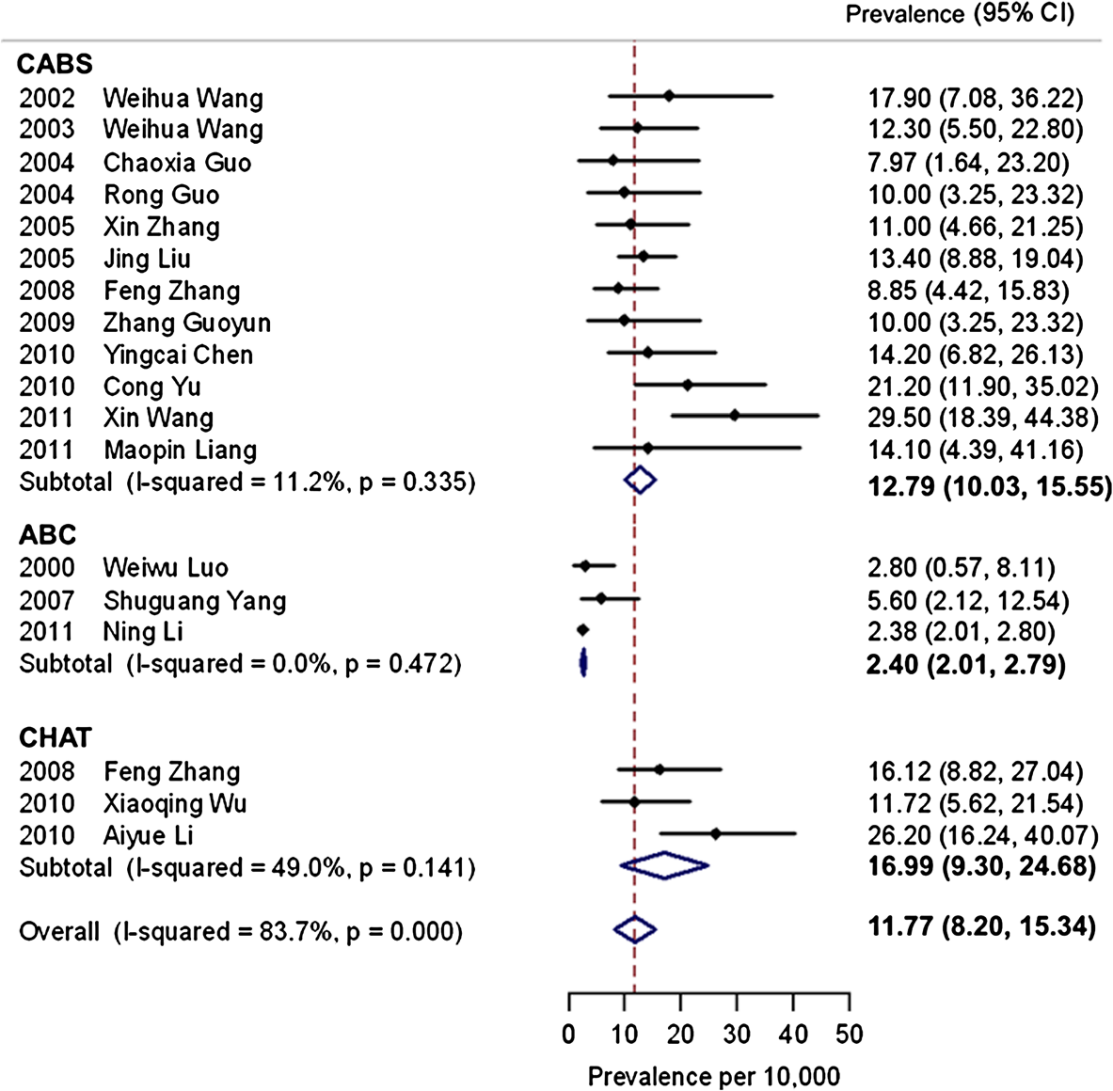
**Prevalence estimates of childhood autism and screening instruments (n = 18).** Dots indicate prevalence estimates in reviewed studies. Horizontal lines indicate 95% CIs. Vertical line indicates pooled prevalence estimate in the meta-analysis. The heterogeneity among studies using the CABS and ABC is low. The pooled prevalence estimates of studies using the ABC as the screening instrument is the lowest, while those using the CHAT reported the highest prevalence. ABC, Autism Behavior Checklist; CABS, Clancy Autism Behavior Scale; CHAT, Checklist for Autism in Toddlers; CIs, confidence intervals.

The pooled prevalence estimate increased over time. Between the years 2000 and 2004, the pooled prevalence estimate was 8.5 per 10,000 (range: 3.0, 13.9), which increased to 10.3 per 10,000 (range: 7.2, 13.4) between 2005 and 2009. The estimate was the highest in the years 2010 to 2011 at 16.4 per 10,000 (range: 7.0, 25.7). The prevalence estimates were higher when using the CABS (12.8 per 10,000) and the CHAT (17.0 per 10,000) as screening instruments, rather than using the ABC (2.4 per 10,000). The heterogeneity was very low among studies using the ABC (*I*^2^= 0.0%) (Figure 3).

Only the studies that conducted both screening and diagnostic assessment for childhood autism were examined using meta-regression, since the recommended minimum number of studies for inclusion in a meta-regression analysis is ten [66]. There was a significant association between the use of screening instruments and the prevalence of childhood autism (Table 2). The prevalence estimates for childhood autism in studies using the ABC as the screening instrument was 79% lower than those studies using the CABS (odds ratio: 0.21; 95% CI: 0.11, 0.38; *P* <0.001). The prevalence estimates from studies using the CHAT were 25% higher than those using the CABS (odds ratio: 1.25; 95% CI: 0.71, 2.21), but the CI was wide and included 1.0.

In three age groups (<4, 4, >4 years old), the prevalence estimates in studies with children older than 4 years old were significantly lower than estimates in younger children (odds ratio: 0.32; 95% CI: 0.16, 0.68). However, this result is dependent on three very large studies. If these three studies with the largest sample size were excluded, this association was not observed (*P*= 0.33). No significant association was observed between the prevalence estimate and group sample size (≤5000, 5000 to 7500, >7500). No other covariates were found to have a significant association with the prevalence estimates.

Among 18 studies focusing on childhood autism in mainland China, the different choice of screening instruments explained 77% of the between-study variance (*R*^2^ = 77%, *I*^2^= 45%) and the age group of the children explained 56% (*R*^2^= 56%, *I*^2^ = 71%).

A meta-regression model was constructed which included screening instrument and age group (Table 2). This model explained much of the heterogeneity between the studies (*R*^2^ = 81%, *I*^2^= 44%). In this model, after adjusting for the age group, the odds ratio for ASC in studies using the ABC as the screening instrument was 0.29 compared with the CABS (95% CI: 0.12, 0.69; *P* = 0.009), whereas studies using the CHAT had higher rates (odds ratio: 1.79; 95% CI: 0.70, 4.55; *P* = 0.20). After adjusting for the screening instrument, age group no longer showed a significant effect.

## Discussion

Among reviewed studies, the covariate most strongly associated with variation in the prevalence estimates for childhood autism was the choice of screening instrument. The association between screening instrument and prevalence estimates has been investigated in Western studies [67-69]. The ABC and CABS (which were developed in the 1980s from the West) were introduced to Chinese autism research much earlier than the CHAT. In reviewed studies, the studies using the ABC as the screening instrument reported the lower prevalence estimates, while studies using the CHAT reported the higher estimates for childhood autism. However, there have been only three studies using either the ABC or the CHAT to date. The comparison of the effect of screening using the ABC or the CHAT on the prevalence estimates is therefore limited. In this review, the age group of the children screened was also found to be strongly associated with the prevalence estimate. Fifteen studies focused on children aged between 2 to 6 years, six studies examined children aged between 6 and 14, and only one study investigated the population older than 14 years old. Most of the reviewed studies included children who were as young as 2 to 3 years old. However, as the average age of diagnosis of ASC is suggested to be 41 to 60 months [70], it was not unexpected that the prevalence estimate of children in this age range would be relatively lower. However, this association disappeared when adjusting for the screening instrument. This may be due to the fact that recently developed screening instruments have specifically determined age ranges, while older measures included participants with a wider age range. After adjusting for age group, the prevalence estimates for childhood autism generated from studies using the ABC as the screening instrument was 70% lower than those using the CABS, and the prevalence estimates in studies using the CHAT was 80% higher than those using the CABS.

The multi-regression model including the age group and screening instrument explained the most among-study variation in studies of childhood autism. The effect of screening instrument was significant. This finding suggested that it may be possible that the adoption of screening instruments influenced the prevalence estimates. The studies using the more recently developed screening instrument (the CHAT) reported higher prevalence than studies using other instruments (the CABS and ABC). The CHAT is designed to screen children for autism spectrum, and the CABS and ABC are developed to detect children with one subtype of ASC, childhood autism. This may be one of the reasons why the prevalence estimates from studies using the CHAT were higher than the CABS and ABC. In addition, half of the reviewed studies did not use any diagnostic instrument and the rest used the CARS as the diagnostic instrument. There was a lack of comparability in the confirmation of case status between Chinese and Western studies. As there were only a limited number of studies using the CHAT or the ABC, further research needs to be conducted to investigate whether this effect still exists when using standardized diagnostic instruments for case confirmation using different screening instruments in the same population. It is important to note that the CHAT misses as many cases as it detects and that a revised version of this instrument, the Q-CHAT, is being evaluated to improve the instrument (Allison et al, 2008).

In addition, there are other differences between studies in developed countries and the reviewed studies. These differences include the following factors. 1. Population characteristics, due to missing information of the target population it is difficult to evaluate the generalizability of the sample for a whole area in mainland China and make comparisons to other countries. In addition, service development for special education and healthcare systems were different in mainland China, Hong Kong and Taiwan. 2. Administration of screening by face-to-face interview is not common in studies in developed countries; many identified studies in this review were based on samples from the stratified general population, while large population-based studies in developed countries used whole population distribution of a screening questionnaire [6]. Screening using two instruments is not common in studies in developed countries and which instrument was administrated first was not clear in these reviewed studies. If a second screening test is applied only to screen positives following the first screening, it is generally considered to increase the specificity by reducing the false-positives compared with a single test [71]. If the two screenings were done simultaneously, this might lead to higher sensitivity, since the children that had been missed by the first screening instrument may have been identified to be at risk for ASC by the second screening instrument [72]; the cut-off of the same screening instruments varied among studies. 3. In the diagnostic phase, four prospective studies considered the screening results to be the diagnostic results; standardized diagnostic instruments were not adopted in reviewed studies; information on the reliability and quality control of the diagnostic process was generally lacking and assessors were not blind to the screen status of the children when making a diagnostic evaluation; and children whose screen results were negative were not given a diagnostic assessment.

Despite the differences discussed above, other factors related to autism in the Chinese population should also be considered when examining prevalence estimates. For example, one factor might be the awareness and recognition of ASC among clinicians. One study investigated awareness among Chinese community physicians, comprehensive hospital pediatricians and parents who were referred to the hospital in Wuxi city [36]. Results indicated parents were the most knowledgeable about the diagnosis of autism compared to pediatricians and physicians. A recent review on healthcare and service provision of ASC in mainland China suggested lower awareness and lack of training in diagnosis of ASC among Chinese clinicians [73]. Another factor might be the lack of knowledge and acceptance of ASC among Chinese parents. Studies on service provision of ASC in mainland China found an unwillingness to accept the diagnosis of ASC among Chinese parents [74]. This may also influence the case identification of prevalence studies since it was mainly the parents who filled in screening and diagnostic questionnaires. A third factor might be the potential cultural influence. People with disabilities usually experience stigma, stemming from within and outside the family influenced by societal beliefs [75]. As autism is a condition categorized into the discipline of psychiatry, many informants in this sample (especially the older generation) considered it at best as not good, and at worst as very bad. Previous studies reported that family members may feel ashamed and embarrassed by their children which can trigger stigma, which can also lead to the difficulty in acceptance of the diagnosis of ASC [76]. Meanwhile, the recognition of autistic features in one culture may be different from another, since each culture has a specific set of behavioral norms and expectations, which are not necessarily the same as the others [29]. Thus, the interpretation of behavioral descriptions on screening and diagnostic instruments can be different among cultures. This may lead to possible differences in the recognition of autistic behaviors between Chinese and Western populations. Previous studies have suggested possible different perceptions of eye contact [29] and the age of speaking among boys [77]. Therefore, although it was not possible to investigate these factors in this review, the potential effects of these factors towards prevalence estimation should not be ruled out when comparing between Chinese and Western populations.

There are several limitations of this review. First, the studies reviewed were selected from two English and two Chinese databases, and no other databases were searched. It is possible that studies that were published in Master’s or doctoral theses in Hong Kong or Taiwan were not included and papers that were not published in mainstream journals were not identified, which may have reported different results. However, the four databases were searched systematically using a consistent approach with a second attempt of one-by-one checking. Thus, it is unlikely that the reviewed papers are biased with respect to prevalence estimates reported. The number of studies included in the meta-analysis was limited, with only 18 for childhood autism and seven for ASC. Meta-regression of studies for ASC was not conducted due to the limited number of available studies. Due to the limited number of studies available for regression analysis, the potential association between the age group and the choice of screening instrument was not further investigated in this review. Thus, the generalization of the results is limited and the interpretations of these results need to be considered carefully. There were a limited number of studies conducted in Hong Kong and Taiwan. Due to the differences among regions, caution should be employed when applying the results from mainland China to Hong Kong and Taiwan. The coding approach of covariates may have affected the detected association with prevalence estimates, such as the approach of categorizing the diagnostic criteria and using the age groups. Since information on the process of screening and diagnosis was often missing, an assumption was made about the participation rate in the assessment phase. It would be helpful to have further information about the details where this information was missing. Only the impact of quantifiable covariates on prevalence estimates was assessed in this review. Potential qualitative influences on prevalence such as public awareness and the recognition of ASC were not included.

## Conclusion

This review revealed major differences in research methodology for estimating prevalence between the developed countries and mainland China, Hong Kong and Taiwan. In the future, in order to make comparisons between studies cross-culturally, it would be valuable to validate more recently published screening instruments for ASC used in Western countries in these three areas. Standardized diagnostic instruments including the ADOS [78] and ADI-R need to be adapted and validated in the Chinese population to make robust comparison possible. The Chinese versions of these two instruments have been approved by the publisher, Western Psychological Services (WPS). More recently, in 2012, a new updated version of the Chinese ADI-R has been finalized by the WPS. Three screening instruments have been validated in the Chinese population in Taiwan including the Social Communication Questionnaire (SCQ) [79], the Social Responsiveness Scale (SRS) [80] and the Autism Spectrum Quotient (AQ) [81]. Another screening instrument, the Childhood Autism Spectrum Test (CAST) [82] has been validated in mainland China. Since the CCMD was developed and only used in mainland China, a more universal and standardized diagnostic process for ASC should be adopted for autism research in Chinese populations. Prospective population-based epidemiological studies of ASC need to be conducted in mainland China, Hong Kong and Taiwan using methods which are comparable across the Chinese population and with the rest of the world.

## Abbreviations

ADI-R: Autism Diagnostic Interview-Revised; ADOS: Autism Diagnostic Observation Schedule; AQ: Autism Spectrum Quotient; ASDASQ: Autism Spectrum Disorder in Adults Screening Questionnaire; CARS: Childhood Autism Rating Scale; CAST: Childhood Autism Spectrum Test; CCMD: Chinese Classification of Mental Disorders; CCMD-2-R: Chinese Classification of Mental Disorders 2nd edition, revised; DSM: Diagnostic and Statistical Manual of Mental Disorders; DSM-III-R: Diagnostic and Statistical Manual of Mental Disorders 3rd edition, revised; DSM-IV: Diagnostic and Statistical Manual of Mental Disorders 4th edition; ICD: International Classification of Diseases; ICD-10: International Classification of Diseases 10th revision; ICD-9: International Classification of Diseases 9th revision; MAA: Multidisciplinary multiagency assessment; NAPC: National Autism Plan for Children; PDD-NOS: Pervasive developmental disorder not otherwise specified; SCQ: Social Communication Questionnaire; SRS: Social Responsiveness Scale; WPS: Western Psychological Services.

## Competing interests

The authors declare that they have no competing interests.

## Authors’ contribution

XS had the idea for the study, searched and extracted the data, and wrote the paper under the supervision of CB, SBC and CA. FM and SS provided advice on analytical approaches. All authors contributed to the data interpretation, and have seen and approved the final version. All authors read and approved the final manuscript.

## Supplementary Material

Additional file 1 Diagnostic criteria for childhood autism in CCMD-2-R. Click here for file

Additional file 2 Search strategy. Click here for file

Additional file 3 Inclusion criteria. Click here for file

Additional file 4 Description epidemiology of the population studied in reviewed papers. Click here for file

Additional file 5 Methodology of screening for case identification in reviewed studies. Click here for file

Additional file 6 Methodology of diagnostic assessment for case confirmation in reviewed studies. Click here for file

Additional file 7 Prevalence estimates in reviewed studies. Click here for file
