# Supplementary material for: Prevalence of autism in mainland China, Hong Kong and Taiwan: a systematic review and meta-analysis
Source: Mol Autism. 2013 Apr 9;4:7. doi: 10.1186/2040-2392-4-7 (PMC3643868; doi:10.1186/2040-2392-4-7)
Supplement: Additional file 1 — Diagnostic criteria for childhood autism in CCMD-2-R. [file 2040-2392-4-7-S1.doc]

**Additional file 1. Diagnostic criteria for Childhood Autism in CCMD-2-R**

(1) The onset is generally by three years old.

(2) Impairment in social interaction by at least two of the following symptoms:

a) Extremely lonely, obvious lack of social emotional reactions, does not have normal communication of emotion with others (including parents);

b) Cannot use eye gaze, facial expression, gestures or hand gestures to communicate;

c) Cannot establish relationship with peers;

d) Lack of interests in group games, cannot have fun or emotional resonance through group activities;

e) Does not seek for help or comfort when has illness or difficulties; Does not offer support or comfort to others when others encounter problems.

(3) Language impairments by at least two of the following symptoms:

a) Language delay or cannot vocalize, such as cannot mimic others words , but may show attempts to use gestures or other formats to substitute verbal communication;

b) Stereotyped and repetitive words which are unrelated to the current environment or ongoing activities;

c) Oddness in the intonation, speed, rhythm or tone of speech;

d) Obvious impairments in understanding;

e) Normal development of language before two years old, but delayed development of language after two, or even cannot speak.

(4) Peculiar interests or activities by at least two of the following symptoms:

a) Stereotyped or circumscribed interests (for example preoccupations with date, advertisements, weather reports etc.);

b) Special attachment to specific objectives;

c) Specific compulsions and rituals;

d) Stereotyped and repetitive movements or gestures;

e) Specific interests in parts of object that is clearly separate from that which is ordinarily accepted (such as smell, feel of the surface, certain noise etc.);

f) Resistance to any changes in personal life environment.

(5) Exclude the following diagnoses: Childhood Schizophrenia, Asperger Syndrome, Heller Syndrome, and Rett¡¯s Syndrome.

**Diagnostic criteria for Childhood Autism in CCMD-3**

(I) Definition: Childhood Autism is one of the pervasive developmental disorders, more common in boys with early childhood onset. Individuals with Childhood Autism have impairments in social communication, circumscribed interests and stereotyped behaviours. Approximately three quarters of affected children have obvious mental retardation, and some children show special talents although their intelligence are generally below the average.

(II) Diagnostic criteria:

At least seven items in the following three categories, of which at least two items in category 1, and at least one item each in category 2 and 3.

1. Impairments in social interaction, at least two items:

1) Lack of interests in group games, alone, cannot have fun or emotional resonance through group activities;

2) Lack of communication technique, failure to develop peer relationships, such as only communicate by dragging, pushing or hugging peers;

3) Self-entertained, lack of interaction with others in surroundings, lack of social observation and emotional reciprocity (including no appropriate reaction to the existence of parents);

4) Marked impairments in the use of eye-to-eye gaze, facial expression, body posture, and gestures to regulate social interaction;

5) Failure to play social games or imitative games (such as cannot play with figures for a familiar family event);

6) When discomfort or unhappy, does not seek for sympathy or comfort, and does not show sympathy or offer to comfort to others when others are discomfort or unhappy.

2. Impairments in verbal communication, mainly in the functional usage of language:

1) Delay in spoken language or cannot use language to express him/herself, does not use gestures, or imitate other people for communication either;

2) Marked impairments in language understanding, usually cannot understand instructions or orders, failure to show needs or difficulties, seldom ask questions and lack of response to what the others say;

3) Refuse to change repetitive and stereotyped movements or gestures, otherwise he/she will be irritated and restless;

4) Persistent preoccupation with parts of objects such as a piece of paper, a smooth piece of cloth, wheels of toy cars and so on, usually shows great satisfaction from these parts of objects;

5) Compulsive adherence to specific, non-functional routines or rituals.

(III) Criteria for severity: impairments in social interaction.

(IV) Onset: generally before three years old.

(V) Exclusion criteria: exclude the following diagnoses including Asperger Syndrome, Heller Syndrome, Rett¡¯s Syndrome, Specific receptive language impairments, and Childhood Schizophrenia.
