# Supplementary material for: Prevalence of autism in mainland China, Hong Kong and Taiwan: a systematic review and meta-analysis
Source: Mol Autism. 2013 Apr 9;4:7. doi: 10.1186/2040-2392-4-7 (PMC3643868; doi:10.1186/2040-2392-4-7)
Supplement: Additional file 3 — Inclusion criteria. [file 2040-2392-4-7-S3.doc]

**Additional file 3. Inclusion criteria**

**Box 3: Inclusion criteria of study selection for review**
