# Supplementary material for: Prevalence of autism in mainland China, Hong Kong and Taiwan: a systematic review and meta-analysis
Source: Mol Autism. 2013 Apr 9;4:7. doi: 10.1186/2040-2392-4-7 (PMC3643868; doi:10.1186/2040-2392-4-7)
Supplement: Additional file 4 — Description epidemiology of the population studied in reviewed papers. [file 2040-2392-4-7-S4.doc]

**Additional file 4.** Description epidemiology of the population studied in reviewed papers

| **No** | **Year** | **First author** | **Region** | **City or county** | **Sample size** | **Area** | **Age** | **Sample selection method** | **Sample source** |
| --- | --- | --- | --- | --- | --- | --- | --- | --- | --- |
| 1 | 1987 | Tao [25] | Mainland | Nanjing | 457,200* | Urban | 3-8 | Case counting | Clinical patients |
| 2 | 2000 | Luo [51] | Mainland | Fujian Province | 10,802 | Mixed | 2-14 | Randomized sampling | Stratified general population |
| 3 | 2002 | Wang[30] | Mainland | Changzhou | 3.978 | Urban | 2-6 | Randomized sampling | Kindergarten population |
| 4 | 2002 | Ren [43] | Mainland | Tongling | 3,559 | Urban | 3-5 | Whole sample | Stratified general population |
| 5 | 2003 | Wang[52] | Mainland | Changzhou, Yizheng | 7,488 | Mixed | 2-6 | Whole sample | Stratified general population |
| 6 | 2003 | Chang[26] | Taiwan | Taiwan | 660 | Mixed | 15-93 | Case counting | Clinical patients |
| 7 | 2004 | Guo [53] | Mainland | Tianjin | 5,000 | Urban | 0-6 | Whole population | One district population |
| 8 | 2004 | Guo [54] | Mainland | Dingxi | 3,776 | Rural | 2-6 | Randomized sampling | Stratified general population |
| 9 | 2005 | Zhang [55] | Mainland | Tianjin | 7,416 | Urban | 2-6 | Randomized sampling | Stratified general population |
| 10 | 2005 | Zhang [29] | Mainland | Wu Han | 1,305 | Urban | 3-7 | Clustered randomized sampling | Kindergarten population |
| 11 | 2005 | Liu [56] | Mainland | Beijing | 21,866 | Mixed | 2-6 | Clustered probability sampling | Stratified general population |
| 12 | 2007 | Yang [31] | Mainland | Zunyi | 10,412 | Urban | 3-12 | Randomized sampling | Primary school population |
| 13 | 2007 | Wong [22] | Hong Kong | Hong Kong | 4,247,206* | Mixed | 0-14 | Case counting | Population in health system |
| 14 | 2008 | Zhang [21] | Mainland | Wuxi | 8,681 | Urban | 2-3 | Randomized sampling | Stratified general population |
| 15 | 2008 | Zhang [21] | Mainland | Wuxi | 12,430 | Urban | 4-6 | Randomized sampling | Stratified general population |
| 16 | 2009 | Zhang [57] | Mainland | Guiyang | 5,000 | Urban | 0-6 | Clustered probability sampling | Stratified general population |
| 17 | 2009 | Wang [28] | Mainland | Meizhou | 4,156 | Urban | 2-6 | Clustered randomized sampling | Kindergarten population |
| 18 | 2010 | Li [58] | Mainland | Tianjin | 8,006 | Mixed | 1.5-3 | Clustered randomized sampling | Stratified general population |
| 19 | 2010 | Wu [59] | Mainland | Lianyungang | 8,532 | Urban | 0-3 | Clustered randomized sampling | Stratified general population |
| 20 | 2010 | Yu [33] | Mainland | Harbin | 7,059 | Mixed | 2-6 | Clustered probability sampling | Stratified general population |
| 21 | 2010 | Chen [32] | Mainland | Daqing | 7,034 | Mixed | 2-6 | Clustered randomized sampling | Stratified general population |
| 22 | 2011 | Wang [27] | Mainland | Guangzhou | 7,500 | Urban | 2-6 | Clustered randomized sampling | Kindergarten population |
| 23 | 2011 | Liang [60] | Mainland | Maoming | 2,485 | Urban | 3-6 | Clustered randomized sampling | Kindergarten population |
| 24 | 2011 | Li [24] | Mainland | National | 616,940 | Mixed | 0-17 | Clustered probability sampling | Stratified general population |
| 25 | 2011 | Chien [23] | Taiwan | Taiwan | 372,642* | Mixed | 0-17 | Case counting | Population in health system |
