# Supplementary material for: Prevalence of autism in mainland China, Hong Kong and Taiwan: a systematic review and meta-analysis
Source: Mol Autism. 2013 Apr 9;4:7. doi: 10.1186/2040-2392-4-7 (PMC3643868; doi:10.1186/2040-2392-4-7)
Supplement: Additional file 5 — Methodology of screening for case identification in reviewed studies. [file 2040-2392-4-7-S5.doc]

### Additional file 5. Methodology of screening for case identification in reviewed studies

| **No** | **Year** | **First author** | **Sample**  **screened** | **Screen**  **methods** | **Screen**  **instruments** | **Cut-off of**  **screening (range)** | **Response rate** | **Screen informants** | **Further**  **screen (Yes/No)** |
| --- | --- | --- | --- | --- | --- | --- | --- | --- | --- |
| 1 | 1987 | Tao [25] | Clinical services | R | N/A | N/A | N/A | Clinicians | No |
| 2 | 2000 | Luo [51] | General population | QI | ABC | 31 | 100% | Parents & Researchers | No |
| 3 | 2002 | Wang[30] | General population | QI | CABS* | 7 (0-14) | 98.3% | Clinicians | No |
| 4 | 2002 | Ren [43] | Kindergarten | QI | CABS | 14 (0-28) | 99.1% | Researchers | No |
| 5 | 2003 | Wang[52] | General population | QI | CABS | 7 (0-14) | 98.08% | Clinicians | No |
| 6 | 2003 | Chang[26] | Clinical patients | C | ASDASQ | 5 (0-9) | 100% | Clinicians | No |
| 7 | 2004 | Guo [53] | Whole population | QI | CABS | 7 (0-14) | 99.1% | Researchers | No |
| 8 | 2004 | Guo [54] | General population | QI | CABS | 7 (0-14) | 100% | Clinicians | No |
| 9 | 2005 | Zhang [55] | General population | QI | CABS | 7 (0-14) | 99% | Researchers | Yes- CARS |
| 10 | 2005 | Zhang [29] | Kindergarten | QI | CABS | 14 (0-28) | 100% | Researchers | No |
| 11 | 2005 | Liu [56] | General population | QI | CABS | 7 (0-14) | 100% | Researchers | Yes--CARS |
| 12 | 2007 | Yang [31] | General population | QI | ABC | 31 | 100% | Researchers | No |
| 13 | 2007 | Wong [22] | Clinical services | R | N/A | N/A | N/A | Clinicians | No |
| 14 | 2008 | Zhang [21] | General population | QI | CHAT (2-3 yrs) | CHAT: N/A | 100% | Clinicians | No |
| 15 | 2008 | Zhang [21] | General population | QI | CABS (4-6 yrs) | CABS: 14 (0-28) | 100% | Clinicians | No |
| 16 | 2009 | Zhang [57] | General population | QI | CABS | 7 (0-14) | 99.98% | Clinicians | No |
| 17 | 2009 | Wang [28] | Kindergarten | QI | CABS | 14 (0-28) | 100% | Researchers | No |
| 18 | 2010 | Li [58] | General population | QI | CHAT | Failed 2 domains | 92.99% | Clinicians | Yes-ABC |
| 19 | 2010 | Wu [59] | General population | QI | CHAT | N/A | 100% | Researchers | No |
| 20 | 2010 | Yu [33] | General population | Q | CABS | 7 (0-14) | 89.7% | Parents | Yes-ABC |
| 21 | 2010 | Chen [32] | General population | Q | CABS | 7 (0-14) | 98.78% | Parents | Yes-ABC |
| 22 | 2011 | Wang [27] | Kindergarten | QI | CABS | 14 (0-28) | 87.8% | Clinicians & researchers | No |
| 23 | 2011 | Liang [60] | Kindergarten | QI | CABS | 14 (0-28) | 100% | Researchers | No |
| 24 | 2011 | Li [24] | General population | QI | ABC | N/A | N/A | Clinicians | No |
| 25 | 2011 | Chien [23] | Taiwan | R | N/A | N/A | N/A | Clinicians | No |

Screen methods: R=Records; QI= Questionnaire based interview; C= Clinical referral; Q=Questionnaire distribution. ABC: Autism Behaviour Checklist; CABS: Clancy Autism Behavioural Scale; ASDASQ: Autism Spectrum Disorder in Adults Screening Questionnaire; CHAT= Checklist for Autism in Toddlers; CARS: Childhood autism Rating Scale. *CABS has two scales: one is 1or 2 for each item and the other is 0,1or 2.
