# Supplementary material for: Prevalence of autism in mainland China, Hong Kong and Taiwan: a systematic review and meta-analysis
Source: Mol Autism. 2013 Apr 9;4:7. doi: 10.1186/2040-2392-4-7 (PMC3643868; doi:10.1186/2040-2392-4-7)
Supplement: Additional file 6 — Methodology of diagnostic assessment for case confirmation in reviewed studies. [file 2040-2392-4-7-S6.doc]

### Additional file 6. Methodology of diagnostic assessment for case confirmation in reviewed studies

| **No** | **Year** | **First author** | **P/R** | **Assessment tool** | **Diagnostic criteria** | **IQ or other tests** | **Assessment**  **informants** | **Clinical**  **judgement** | **Assessment**  **Agreement** |
| --- | --- | --- | --- | --- | --- | --- | --- | --- | --- |
| 1 | 1987 | Tao [25] | R | N/A | Rutter | N/A | Clinicians | Yes | N/A |
| 2 | 2000 | Luo [51] | P | N/A | CCMD-2-R, DSM-III-R | N/A | Researchers | Yes | 0.6-1.0 |
| 3 | 2002 | Wang[30] | P | CARS | CCMD-2-R | Gaseel, PEP | Clinicians | Yes | 0.81-0.90 |
| 4 | 2002 | Ren [43] | P | N/A | N/A | N/A | N/A | No | N/A |
| 5 | 2003 | Wang[52] | P | CARS | CCMD-2-R | Gaseel, PEP | Clinicians | Yes | N/A |
| 6 | 2003 | Chang[26] | P | N/A | DSM-IV | N/A | Clinicians | Yes | N/A |
| 7 | 2004 | Guo [53] | P | CARS | CCMD-2-R | PEP | Researchers | Yes | ≥0.95 |
| 8 | 2004 | Guo [54] | P | CARS | DSM-IV | PEP | Clinicians | Yes | 0.998 |
| 9 | 2005 | Zhang [55] | P | CARS | DSM-IV | DDST, Gaseel | Clinicians | Yes | ≥0.95 |
| 10 | 2005 | Zhang [29] | P | N/A | N/A | N/A | N/A | No | N/A |
| 11 | 2005 | Liu [56] | P | CARS | DSM-IV | N/A | Clinicians | Yes | 1.0 |
| 12 | 2007 | Yang [31] | P | N/A | DSM-IV | N/A | Researchers | Yes | N/A |
| 13 | 2007 | Wong [22] | R | CARS, ADI-R | DSM-IV | N/A | Clinicians | Yes | N/A |
| 14 | 2008 | Zhang [21] | P | CARS | DSM-IV | DDST | Clinicians | Yes | ≥0.95 |
| 15 | 2008 | Zhang [21] | P | CARS | DSM-IV | DDST | Clinicians | Yes | ≥0.95 |
| 16 | 2009 | Zhang [57] | P | CARS | CCMD-2-R | Gaseel, PEP | Clinicians | Yes | 0.81-0.90 |
| 17 | 2009 | Wang [28] | P | N/A | N/A | N/A | N/A | No | N/A |
| 18 | 2010 | Li [58] | P | CARS | DSM-IV | N/A | Clinicians | Yes | 0.98 |
| 19 | 2010 | Wu [59] | P | CARS | DSM-IV | DDST | Clinicians | Yes | ≥0.95 |
| 20 | 2010 | Yu [33] | P | N/A | DSM-IV | N/A | Clinicians | Yes | N/A |
| 21 | 2010 | Chen [32] | P | CARS | DSM-IV | N/A | Clinicians | Yes | ≥0.98 |
| 22 | 2011 | Wang [27] | P | N/A | DSM-IV | N/A | Clinicians | Yes | N/A |
| 23 | 2011 | Liang [60] | P | N/A | DSM-IV, ICD-10 | N/A | Clinicians | Yes | N/A |
| 24 | 2011 | Li [24] | P | N/A | ICD-10 | N/A | Clinicians | Yes | N/A |
| 25 | 2011 | Chien [23] | R | N/A | ICD-9 | N/A | Clinician | Yes | N/A |

DDST: Denver Developmental Screening Test; Gesell: Gesell Developmental Scale; P/R: Prospective/Retrospective.
