# Supplementary material for: Prevalence of autism in mainland China, Hong Kong and Taiwan: a systematic review and meta-analysis
Source: Mol Autism. 2013 Apr 9;4:7. doi: 10.1186/2040-2392-4-7 (PMC3643868; doi:10.1186/2040-2392-4-7)
Supplement: Additional file 7 — Prevalence estimates in reviewed studies. [file 2040-2392-4-7-S7.doc]

### Additional file 7. Prevalence estimates in reviewed studies

| **No** | **Year** | **First author** | **Screen**  **positives** | **Final No**  **of cases** | **Childhood autism** | | **Other ASC** | | **All ASC** | |
| --- | --- | --- | --- | --- | --- | --- | --- | --- | --- | --- |
|  |  |  |  |  | **Prevalence/SE**  **(per 10,000)** | **Gender ratio**  **(Male : Female)** | **Prevalence/SE**  **(per 10,000)** | **Gender ratio**  **(Male : Female)** | **Prevalence/SE**  **(per 10,000)** | **Gender ratio**  **(Male : Female)** |
| 1 | 1987 | Tao [25] | - | 10 | 0.32 (0.08) | 6.5:1 | - | - | - | - |
| 2 | 2000 | Luo [51] | 39 | 3 | 2.8 (1.60) | 4.1:1 | - | - | - | - |
| 3 | 2002 | Wang[30] | N/A | 7 | 17.9 (211.94) | 2.5:1 | - | - | - | - |
| 4 | 2002 | Ren [43] | 89 | N/A | 250 (2.31) | 8:9 | - | - | - | - |
| 5 | 2003 | Wang[52] | 16 | 9 | 12.3 (4.05) | 2:1 | - | - | - | - |
| 6 | 2003 | Chang[26] | N/A | 4 | - | - | - | - | 60 (30.06) | N/A |
| 7 | 2004 | Guo [53] | 5 | 5 | 10 (4.47) | All male | - | - | - | - |
| 8 | 2004 | Guo [54] | 18 | 3 | 7.97 (4.59) | N/A | - | - | - | - |
| 9 | 2005 | Zhang [55] | 16 | 8 | 11 (3.85) | 7:1 | - | - | - | - |
| 10 | 2005 | Zhang [29] | 26 | N/A | 19.9 (2.47) | 2.71:1 | - | - | - | - |
| 11 | 2005 | Liu [56] | 54 | 16 | 13.4 (2.47) | N/A | 1.92 (0.94) | N/A | 15.3 (2.64) | 1.29:1 |
| 12 | 2007 | Yang [31] | 24 | 6 | 5.6 (2.32) | 5:1 | - | - | - | - |
| 13 | 2007 | Wong [22] | N/A | 682 | - | - | - | - | 16.1 (0.19) | 6.58:1 |
| 14 | 2008 | Zhang [21] | 31 | 14 | 16.1 (4.3) (2-3 yrs) | 3.2:1 | - | - | - | - |
| 15 | 2008 | Zhang [21] |  | 11 | 8.85 (2.7) (4-6 yrs) |  | - | - | - | - |
| 16 | 2009 | Zhang [57] | 5 | 5 | 10 (4.47) | 4:1 | - | - | - | - |
| 17 | 2009 | Wang [28] | 81 | N/A | 19.5 (6.84) | 2.68:1 | - | - | - | - |
| 18 | 2010 | Li [58] | 432 | 21 | 26.2 (5.71) | 4.25:1 | - | - | - | - |
| 19 | 2010 | Wu [59] | 7 | N/A | 8.2 (3.10) | 3.5:1 | - | - | - | - |
| 20 | 2010 | Yu [33] | 115 | 16 | 21.2 (5.47) | N/A | 1.42 (1.42) | N/A | 22.7 (5.66) | 7:1 |
| 21 | 2010 | Chen [32] | 44 | 17 | 14.2 (4.49) | N/A | 9.95 (3.76) | N/A | 24.2 (5.86) | 3.25:1 |
| 22 | 2011 | Wang [27] | 746 | 46 | 29.5 (6.26) | 2.6:1 | 45.9 (7.81) | 27:1 | 75.4 (9.99) | 6.6:1 |
| 23 | 2011 | Liang [60] | 35 | 35 | 14.1 (7.53) | 6:1 | - | - | - | - |
| 24 | 2011 | Li [24] | N/A | 77,301 | 2.38 (0.20) | 2.46:1 | - | - | - | - |
| 25 | 2011 | Chien [23] | N/A | N/A | - | - | - | - | 28.7 (0.88) | N/A |
